# Environment as a Risk Factor for Male Infertility

**DOI:** 10.1100/tsw.2001.296

**Published:** 2001-10-18

**Authors:** Luc Multigner, Alejandro Oliva

**Affiliations:** INSERM U435, Université Rennes, France

**Keywords:** infertility, male infertility, environment, sperm, reproductive hormones, pesticides, solvents

## Abstract

Infertility affects 15% of couples in Western countries. Infertility is defined as the inability to conceive after 1 year of attempts without contraception, but it is not synonymous with sterility. Between 30 and 50% of infertile couples are infertile due to male reasons, mainly due to sperm production disorders. Although some risk factors, most of which are infectious, have been identified, there is still much uncertainty about the origins of male infertility.

It was first revealed that environmental factors could affect male reproductive function 30 years ago, when pesticide manufacturers and agricultural workers who came into contact with the nematocide, 1-2-dibromo-3-chloropropane, were shown to suffer from severely impaired spermatogenesis, leading to infertility[1,2]. Since this time, many widely used chemicals have been demonstrated to be toxic to male reproductive function in animal models[3]. However, their effects remain largely unexplored in humans, except for those of a few chemicals[4,5,6,7]. Preliminary studies in Belgium[8], U.S.[9] and the Netherlands[10] have provided evidence that occupational exposure to solvents or pesticides may be associated with poor sperm characteristics in infertile men. Awareness has been further stimulated by reports suggesting that sperm production has declined over the last 50 years and that this phenomenon is related to increased amounts of industrial and agricultural pollution[11,12,13].

A study was recently carried out in the Santa Fe and Entre Rios provinces of Argentina[14] . This area, which is one of the most fertile farmland zones in the world, is characterized by intensive agricultural and industrial activity. The study evaluated the relationship between exposure to environmental agents and seminal characteristics, and the concentration of reproductive hormones in male partners of infertile couples attending infertility clinics between 1995 and 1998. For each individual, information was obtained on basic demographic, medical, surgical, reproductive and occupational history, and lifestyle. A complete physical and andrological examination was carried out and men gave a semen specimen and blood sample. They were divided into five groups according to environmental exposure: nonexposed, exposed to pesticides, exposed to solvents, exposed to heat, and exposed to more than one of these.

A multiple logistic regression analysis was used to produce the odds ratio (OR) and the 95% confidence intervals (95% CI) for the association between seminal characteristics and exposure variables adjusted for confounding factors. Exposure to pesticides significantly increased the risk of having a sperm concentration of below 1 million/ml (OR 3.0, 95% CI 1.2–7.4), a total number of sperm per ejaculate below 3 million (OR 2.7, 95% CI 1.1–6.7), less than 50% motile spermatozoa (OR 4.5, 95% CI 1.8–11.5), and less than 30% of spermatozoa with normal morphology (OR 2.1, 95% CI 1.0–4.8). These associations were higher in men who were frequently exposed than in men who were only occasionally exposed. Exposure to solvents also increased the risk of having poor sperm characteristics, particularly poor sperm motility (OR 3.1, 95% CI 1.0–9.5). Men exposed to pesticides presented elevated levels of serum oestradiol and an increased oestradiol/testosterone ratio compared to nonexposed men. Men exposed to solvents also had low concentrations of luteinizing hormone.

This study showed that exposure to pesticides and solvents is associated with much lower threshold sperm values than those that are considered to be the limits for male fertility. All of these associations were stronger in men who had never fathered a child (primary infertility) than in men who had previously fathered a child (secondary infertility). This may be because environmental factors worsen the effects of predisposing genetic or medical factors for infertility, as is frequently observed in men with primary infertility. Pesticides and solvents may act directly on the testes, where they target germ cells, Sertoli cells, or Leydig cells[3]. However, other mechanisms should be considered. The increased estrogen/androgen imbalance seen in men exposed to pesticides may be related to the disrupting endocrine activity of some chemicals[15].

Infertility is a major problem for public health. Nowadays, male infertility can be treated in a number of ways, thanks to medically assisted reproduction techniques. However, these expensive approaches raise several ethical questions and their long-term effects on descendants are still poorly known. Due to these difficulties, the best way of answering questions about the risks caused by our environment is to develop real prevention and public health policies. In-depth studies of the environmental risk factors for male infertility must be carried out. However, given the great diversity of chemical substances that men are exposed to on a daily basis, epidemiological studies cannot easily be used to incriminate a given substance. It is therefore also necessary to carry out a detailed toxicological evaluation of these substances to determine whether they are toxic to male reproductive function.
